# Immuno-PET for Clinical Theranostic Approaches

**DOI:** 10.3390/ijms18010057

**Published:** 2016-12-28

**Authors:** Clément Bailly, Pierre-François Cléry, Alain Faivre-Chauvet, Mickael Bourgeois, François Guérard, Ferid Haddad, Jacques Barbet, Michel Chérel, Françoise Kraeber-Bodéré, Thomas Carlier, Caroline Bodet-Milin

**Affiliations:** 1 Nantes-Angers Cancer Research Center (CRCNA), University of Nantes, Inserm UMR 892, 8 quai Moncousu, 44007 Nantes, France; clement.bailly@chu-nantes.fr (C.B.); alain.faivre-chauvet@univ-nantes.fr (A.F.-C.); mickael.bourgeois@nantes.inserm.fr (M.B.); francois.guerard@univ-nantes.fr (F.G.); michel.cherel@univ-nantes.fr (M.C.); francoise.bodere@chu-nantes.fr (F.K.-B.); thomas.carlier@chu-nantes.fr (T.C.); 2 Department of Nuclear Medicine, CHU de Nantes, 1 place Alexis Ricordeau, 44093 Nantes, France; pf7.clery@gmail.com; 3 Groupement d’Intérêt Public Arronax, 1, rue Aronnax, CS 10112, 44817 Saint-Herblain, France; ferid.haddad@univ-nantes.fr (F.H.); jacques.barbet@univ-nantes.fr (J.B.); 4 Department of Nuclear Medicine, Institut de Cancérologie de l’Ouest (ICO)-René Gauducheau, Boulevard Jacques Monod, 44805 Saint-Herblain, France

**Keywords:** immuno-PET, molecular imaging, antibody

## Abstract

Recent advances in molecular characterization of tumors have allowed identification of new molecular targets on tumor cells or biomarkers. In medical practice, the identification of these biomarkers slowly but surely becomes a prerequisite before any treatment decision, leading to the concept of personalized medicine. Immuno-positron emission tomography (PET) fits perfectly with this approach. Indeed, monoclonal antibodies (mAbs) labelled with radionuclides represent promising probes for theranostic approaches, offering a non-invasive solution to assess in vivo target expression and distribution. Immuno-PET can potentially provide useful information for patient risk stratification, diagnosis, selection of targeted therapies, evaluation of response to therapy, prediction of adverse effects or for titrating doses for radioimmunotherapy. This paper reviews some aspects and recent developments in labelling methods, biological targets, and clinical data of some novel PET radiopharmaceuticals.

## 1. Introduction

Recent decades have seen the discovery of oncogenesis and tumor suppressor genes, which along with the progressive deciphering of cellular signal transduction pathways, defined the biological hallmarks of cancer [1,2]. This initiated a new era of cancer therapy by developing the use of targeted molecular therapies in complement to cytotoxic drugs and nonspecific chemotherapy. The two main types of these treatments are monoclonal antibodies (mAbs) [3,4] and tyrosine kinase inhibitors (TKIs) [5,6]. They are designed to interfere specifically with single or multiple key molecular pathways involved in tumorigenesis, fulfilling Ehrlich’s vision of a “magic bullet”, capable of selectively destroying disseminated tumor cells while sparing normal tissues [7].

A key discovery for the production of mAbs was the hybridoma technology, by Köhler and Milstein [8], which allowed unlimited generation of stable mAbs with predetermined specificity. Yet, during their early use, the immunogenicity of these murine mAbs constituted the main obstacle to their therapeutic success. This led to the development of a second-generation of chimeric and humanized mAbs [9]. The first highly specific mAbs targeting the human epidermal growth factor receptor 2 (HER2) or CD20 opened the door to novel therapeutic strategies and represented an impressive step forward. The clinical efficacy of the anti-HER2 trastuzumab in patients with breast cancer and the anti-CD20 rituximab in B non-Hodgkin lymphoma (NHL) demonstrated for the first time that mAbs can be commercialized as powerful therapeutic agents in the fight against cancer [10,11]. Clinical success with rituximab and trastuzumab energized the research of new target membrane proteins in lymphomas and solid tumors. In the meantime, in the 1980s, a few mAbs were labelled for scintigraphic imaging of tumors [12]. It was a promising challenge but the expected success was limited by a poor imaging resolution despite a rather good specificity of the mAbs. Yet, thanks to the development of more sensitive detectors and specific software, along with significant technical advances in the production of positron-emitting radionuclides and their related labelling methods, a broad range of new tracers for the realization of specific imaging [13,14,15,16] were developed in the last decade. In medical practice, the identification of biomarkers will slowly but surely become a prerequisite before any treatment decision, leading to the concept of personalized medicine. Immuno-positron emission tomography (PET), combining the high sensitivity and resolution of a PET camera with the specificity of a mAb, perfectly fits with this approach. Indeed, mAbs labelled with radionuclides represent promising probes for theranostic approaches, offering a non-invasive solution to assess in vivo target expression and distribution and to obtain reliable diagnostic, prognostic and therapeutic information [17]. This overview of targets’ distribution could thus be incorporated into individual treatment strategies before the introduction of potentially expensive or toxic therapies [18]. Moreover, one could imagine a role of immuno-PET to facilitate the development of new drugs by pharmaceutical companies. Radio-labelling potential candidates during early development phases could constitute an effective and rapid solution to monitor their pharmacokinetics and distribution. Once labelled with β- or α-emitters, radiolabelled mAbs targeting relevant molecular markers expressed by different solid tumors or hemopathies can be used for radioimmunotherapy (RIT). This short review provides an overview of the main issues, current use and promising results of immuno-PET in line with the development of personalized medicine.

## 2. Radionuclides

Since the 1990s, mAbs have been labelled with γ-emitting radionuclides, such as ^99m^Tc or ^111^In, and imaged with planar or Single Photon Emission Computerized Tomography (SPECT) cameras. Although informative, these imaging modalities suffered from limited sensitivity and low-spatial resolution, and did not provide reliable quantitative measurements. In this context, due to high sensitivity, improved spatial resolution and signal-to-noise ratios, coupled with the capability to perform accurate quantification, PET has rapidly emerged as a very powerful method for mAbs imaging [19].

Combining mAbs and positron-emitters requires an appropriate match between the biologic half-life of the protein and the physical half-life of the radionuclide to achieve optimal tumor-to-background activity ratios [20,21]. Indeed, intact antibodies have a long residence time (several days) due to slow blood clearance resulting in optimal image contrast and tumor-to-background activity ratios only at prolonged time points after injection. Thus, the use of short half-life ^18^F (*t*_1/2_ = 110 min) or ^68^Ga (*t*_1/2_ = 68 min) is limited to small size molecules such as peptides or small molecular weight proteins such as mAbs fragments that distribute rapidly in the body. On the other side, ^89^Zr (*t*_1/2_ = 78.4 h) and ^124^I (*t*_1/2_ = 100 h) are more suitable for large molecule labelling, such as intact mAbs. ^64^Cu with an intermediate half-life of 12.7 h can be used for labelling a large number of molecules with different sizes. Radionuclides with a long half-life also offer logistic advantages with respect to radiolabelling and transportation outside the production site but also potential disadvantages with respect to the radiation burden to the patient, especially when coupled to intact mAbs with long biologic half-lives. Finally, within the scope of a theranostic approach, both the imaging and the therapeutic products can be labelled using the same chemical elements with pairs of β+/β− emitting radionuclides (^124^I/^131^I, ^86^Y/^90^Y, ^64^Cu/^67^Cu, ^44^Sc/^47^Sc).

Several additional considerations must also be taken into account in selecting the appropriate radionuclides [21]. Positron energy range may affect resolution as the positron may travel a significant distance before annihilation: high-energy positron will result in an intrinsic resolution loss. In addition to half-life, existence of concomitant γ emissions will have major effects on the radiation dose to the patient. Finally, other factors to consider include cost and accessibility.

Imaging with intact mAbs typically requires a minimum delay of more than 1 day post-injection before high-contrast images can be obtained and this has spurred the development of imaging agents based on smaller antibody fragments that retain immune-recognition capabilities. These engineered constructs include F(ab′)2 and Fab fragments, diabodies, minibodies, cysdiabodies or affibodies. They offer rapid clearance from systemic circulation, better extravasation and tumor penetration than intact mAbs allowing for imaging on the same day after administration. Immunoconjugates based on these fragments have for the moment demonstrated encouraging significant preclinical [22,23,24] and clinical results [25,26].

## 3. Labelling Techniques

Radionuclides can either be directly conjugated to a mAb or attached indirectly through a linker, depending on their intrinsic properties. The most commonly used PET imaging radionuclides for immuno-PET belong to two classes: radiohalogens and radiometals.

Radiohalogens—of which the main representative is ^124^I—are usually directly conjugated to the biological vector. The iodination procedure by direct electrophilic substitution on a tyrosine residue is the simplest method for protein labelling [27]. This technique is commonly used and provides satisfactory results with non-internalizing antibodies or peptides. Unfortunately, when internalization occurs, intracellular catabolism of the antibody and dehalogenation result in the clearance of radioiodine from the target tissue, leading to signal loss and unspecific accumulation such as thyroid for iodine. A number of research groups have developed prosthetic or pendant groups to solve this problem [28,29,30], but they are still at the level of preclinical proofs of concepts. Concerning ^18^F, although its ease of production in large quantities, its half-life and the low energy of the emitted positron make it an ideal versatile radionuclide for PET imaging, preparing ^18^F-conjugates require time-consuming and challenging radiosynthesis. In the last decade, despite the fact that direct labelling remains a special challenge, tremendous progress has been made regarding the ^18^F-labelling of biomolecules, and several strategies have been introduced beyond the use of prosthetic groups [31,32]. Moreover, the combination of antibody fragments and ^18^F is particularly attractive as the physical half-life of ^18^F will be well matched to the biologic half-life of the small fragments.

Alternatively, radiometals, such as ^68^Ga, ^64^Cu or ^89^Zr necessitate the use of bifunctional chelating agents such as DOTA (1,4,7,10-tetraazacyclododecane-1,4,7,10-tetraacetic acid). The antibody of interest is first conjugated to the chelating agent through covalent attachment. The chelating agent-modified antibody can then provide sequestration of the radionuclide to yield a stable complex. However, the metallic radionuclides are diverse in terms of their coordination chemistries, and thus, various kinds of chelating agents are required to label with many radionuclides [33,34].

One of the main factors to be considered for the selection of a suitable chelating agent for a specific radionuclide is stability. Another additional critical property to consider is the fate of the radionuclide in case of dissociation, or after antibody catabolism. Ideally, intact conjugates would accumulate and be retained selectively at the tumor site while free or chelated radionuclides would be rapidly excreted via the urinary tract without intracellular retention in the non-target tissues. Yet, this is not the case, and unwanted retention of metallic radionuclides is the rule. For example, a lot of recent preclinical studies reported the use of ^89^Zr and its most commonly used chelator, desferrioxamine (DFO) [35]. Yet, significant bone uptake has been reported in preclinical studies even with purified ^89^Zr-DFO-mAb conjugates [36]. This uptake is explained by the release of free ^89^Zr cations within the body that mineralize into the skeleton. This uptake of free ^89^Zr could be of particular concern in the clinic, and has led several groups to investigate the possibility of developing better chelators for ^89^Zr. Similarly, transmetallation or transchelation phenomena can occur in vivo when the radiopharmaceutical is in competition with metal complexing proteins, such as transferrin or ceruloplasmin leading to unwanted liver and kidney uptakes. This potential drawback has been described in a recent review by Zhou [37] with regard to biological behaviors and distributions of radionuclide-labeled mAbs. To limit these phenomena, as dissociation of the radionuclide is directly associated to this loss in image quality, continued research of better chelation agents has been done in order to improve both selectivity and stability. Chelating agents with very high affinities for metals and kinetics stabilities need to be developed [34].

An alternative approach to provide better tumor-to-background ratios and contrast, is pre-targeted imaging [38,39,40]. In this approach, unlabelled antibodies are used, capable of both binding antigens and radiolabelled small molecular weight ligands. After administration, binding, and clearance of the antibody, the radioactive ligand is injected in a second step to bind to the pre-localized antibody. A variety of pre-targeting techniques have been proposed with good results both for imaging and therapy in preclinical and clinical models.

Very recently, the use of click chemistry has also emerged as a new specialized conjugation method for preparing imaging probes [41,42]. Due to their rapid and highly selective nature, bioorthogonal chemistry reactions represented by the strain-promoted alkyne-azide cycloaddition and inverse electron demand Diels-Alder cycloaddition, are attracting a significant amount of interest in the radiopharmaceutical community [43,44]. Click chemistry has the potential to circumvent many of the limitations of its predecessors and has shown significant promise in a few pioneering preclinical studies.

## 4. Applications

**Figure 1:**
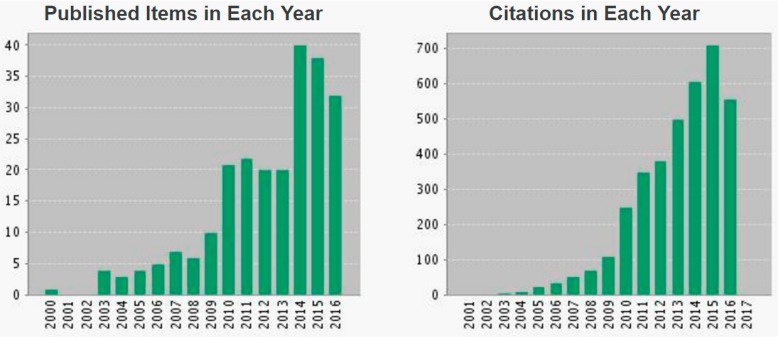
Topic of research on Webofknowledge.com: “immuno-positron emission tomography (PET)”. This report reflects citations to source items indexed within Web of Science Core Collection.

Each patient is unique. The move toward personalized medicine aims to provide the right therapeutic strategy to the right patient at the right dose, at the right time. As previously described, immuno-PET perfectly fits in this concept. Radio-immunoconjugates (RIC) have become prominent in recent developments in oncology because of improvements in mAb technology, and access to novel radionuclides has also improved; together these have increased the clinical attention to immuno-PET in the last 5 years (Figure 1).

mAbs labelled with positron-emitters could indeed have several in vivo uses. They could of course help in molecular diagnosis or be correlated to the prognosis of patients. They could also be considered as companions in theranostic approaches, to non-invasively assess in vivo tumor antigen expression and accessibility, in the context of targeted mAb therapies or RIT. This role in patient selection appears essential and is considered as one the main advantages of immuno-PET, target expression usually being a prerequisite for response. Moreover, if target accessibility is low, therapeutic agents cannot reach the tumor and be effective, even if immunohistochemical expression of receptors on the tumor cells has been assessed. For RIT, immuno-PET could probably help optimizing injected doses too.

The first clinical proof that immuno-PET represents a powerful tool for molecular diagnosis has probably been reported by Divgi et al. [45] in clear cell renal cell carcinoma (ccRCC), with the chimeric antibody cG250 (girentuximab). This mAb reacting against carbonic anhydrase IX (CAIX), a cell-surface antigen over-expressed in the vast majority of ccRCC, was ^124^I-labelled and used in 26 pre-surgical patients with renal masses. In this study, the sensitivity and specificity for ccRCC were respectively 94% and 100%, with a negative predictive value of 90% and a positive predictive value of 100% [45]. These excellent preliminary results have been confirmed in a phase III study (REDECT), demonstrating that PET imaging using ^124^I-labelled girentuximab could accurately and noninvasively assess the presence or absence of ccRCC while avoiding the inherent risks of biopsy [46]. Another study reported a potential interest of CAIX immuno-PET imaging as this protein is also upregulated in many other tumor types and generally correlated with hypoxia. The capability of ^89^Zr-labelled girentuximab to visualize tumor hypoxia was thus assessed, in a preclinical model of human head and neck xenograft tumor. This study reported a significant, positive correlation between ^89^Zr-labelled girentuximab accumulation and CAIX expression on a microscopic level [47]. Even though further clinical studies are warranted, these results suggest an additional potential role for this radioimmunconjugate in defining CAIX-positive hypoxic areas potentially requiring intensified therapy.

Interesting results in diagnostic and prognostic imaging were also obtained with prostate cancer. Prostate-specific membrane antigen (PSMA) is a well-characterized imaging biomarker of prostate cancer directly related to androgen independence, metastasis and progression. One of the first anti-PSMA radioimmunoconjugates is the ^111^In-labelled capromab pendetide (ProstaScint), a mouse monoclonal antibody conjugate that is useful for SPECT imaging [48]. New high affinity antibodies of PSMA for PET imaging have also been developed, such as J415, J533, and J591. The latter is the most extensively studied [49,50], with satisfactory results in accurately targeting bone and soft tissue metastatic prostate cancer sites. A phase I imaging trial with ^89^Zr-labelled J591 in 10 patients with metastatic prostate cancer also demonstrated the ability of this construct to identify metastases, including lesions that were not detected on conventional imaging [51]. Moreover, a recent prospective study in patients awaiting prostatectomy found that a ^89^Zr-labelled conjugate of the J591 antibody was able to identify tumors with a Gleason score of seven or greater [52]. Yet, major disadvantages have been described with these radiolabelled monoclonal antibodies such as the long time from injection for optimal imaging, with several days often required due to poor tumor penetration. To address that issue, multiple small-molecule ligands targeting PSMA have also been explored such as aptamers and PSMA inhibitors of low molecular weight [50].

**Figure 2:**
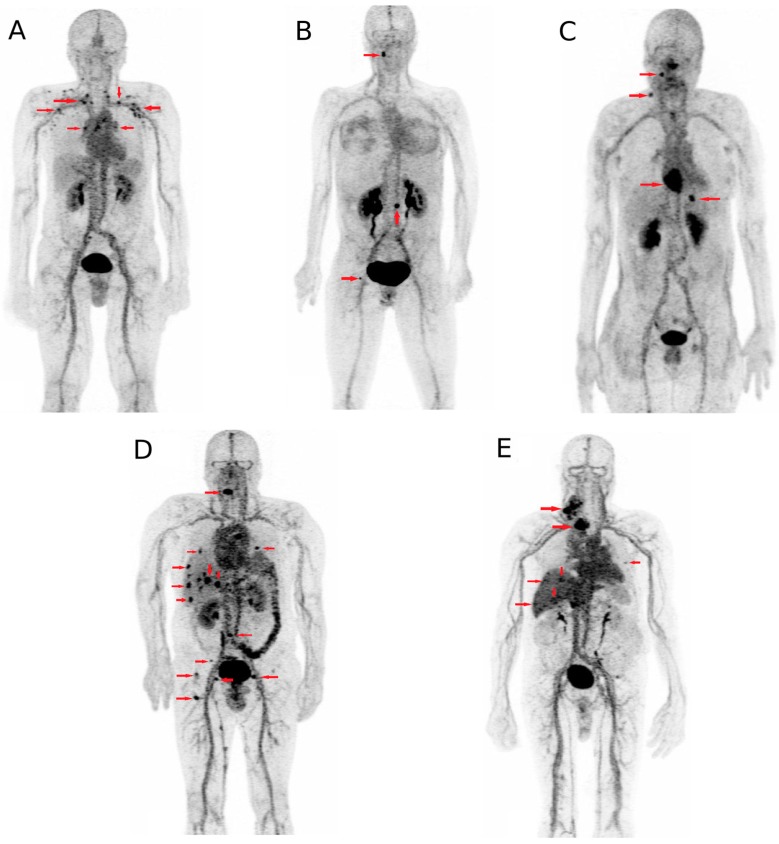
Immuno-PET maximum-intensity-projection images recorded in five patients (one patient of each cohort, **A** to **E**) included in the optimization part of the first-in-human immuno-PET trial using anti-carcinoembryonic antigen CEA bispecific antibody and ^68^Ga-labelled peptide in metastatic medullary thyroid carcinoma. Arrows showed foci considered as pathologic by immuno-PET. (This research was originally published in *JNM*. Immuno-PET using anti-CEA bispecific antibody and ^68^Ga-labelled peptide in metastatic medullary thyroid carcinoma: clinical optimization of the pre-targeting parameters in a First-in Human trial. Bodet-Milin et al. *J. Nucl. Med.* **2016**, *57*, 1505–1511. © by the Society of Nuclear Medicine and Molecular Imaging, Inc., Reston, VA, USA).

The pre-targeting anti-carcinoembryonic antigen (CEA) system using TF2, a humanized trivalent bispecific antibody (BsMab) and IMP288, a radiolabelled hapten have been adapted for immuno-PET in different solid tumor models, using short half-life emitters such as ^18^F or ^68^Ga. In a first human optimization trial testing the feasibility of this system in relapsing medullary thyroid cancer, our team demonstrated that a high-contrast tumor uptake can be obtained using pre-targeted immuno-PET (Figure 2) [40]. Promising results were also obtained in CEA-positive breast cancer patients and another study (ClinicalTrials.gov NCT02587247) is ongoing to evaluate the potential of anti-CEA immuno-PET in colorectal carcinoma.

In the context of personalized medicine, selecting patients before targeted therapy to predict response also appears as an application to consider. Excellent results were obtained with anti-HER2 imaging in breast cancer, and will be further discussed below. Similarly, ^89^Zr-labelled bevacizumab was assessed in seven patients with non-small cell lung cancer and followed by induction therapy combining carboplatin, paclitaxel and bevacizumab. A positive trend without significant correlation was found for tumor uptake and progression-free survival and overall survival after treatment [53]. Similar encouraging results were observed in patients with advanced colorectal cancer who received ^89^Zr-labelled cetuximab followed by treatment with cetuximab [54]. Yet, no association was obseved between tumor accumulation and clinical benefit in other clinical applications such as ^89^Zr-labelled bevacizumab followed by everolimus therapy in patients with neuroendocrine tumors [55] and in patients with recurrent high-grade glioma treated with fresolimumab using ^89^Zr-labelled fresolimumab imaging [56]. Based on these preliminary clinical results, despite some discrepancies and studies with a relatively small numbers of patients, it appears that immuno-PET has a real potential for predicting response to targeted therapy assuming that the radioimmunoconjugate’s biodistribution is representative of the therapeutic mAbs’ biodistribution. The validation of this approach to guide therapeutic strategies requires larger prospective studies demonstrating that stopping or continuing therapy based on imaging results is favorable to patient outcomes compared with standard practice. Moreover, for therapeutic evaluation, if the imaging radiolabelled mAb is the same as the one used for therapy, more investigations will be perhaps needed to determine whether the potential absence of tumor uptake reflects a reduction in target overexpression as a consequence of targeted therapy or impaired targeting explained by partial saturation with unlabelled mAbs [57]. Similarly, optimal dosage of radiolabelled-mAbs might be different in naïve patients than in those undergoing treatment with unlabelled-mAbs [58]. Further randomized multicentric clinical studies are still warranted before immuno-PET could be fully considered as a companion diagnostic test.

One of the most widely used mAbs in clinical practice is trastuzumab, which targets HER2. HER2 plays an important role in breast cancer pathogenesis. Anti-Her2 therapeutic agents are only effective in patients who have HER2-positive breast cancer. In clinical practice, HER2 expression is assessed by immunohistochemistry and technical difficulties can arise when lesions are not accessible to biopsy. Moreover, HER2 status of metastatic lesions can differ from the primary site and discrepancies can appear across lesions within the same patient during the course of the disease. Several reports suggested the negative prognostic impact of these changes in histopathological biomarker profiles between primary and recurrent tumors in breast cancer [59,60,61]. For these reasons, evaluating HER2 status of recurrent breast cancer before therapeutic decisions of targeted therapies seems an essential prerequisite. An anti-HER2 immuno-PET would allow measurement of receptor expression of whole tumors and their metastases, avoiding sampling errors and thus misinterpretation due to intratumoral and interlesional heterogeneity, without the need for repeated invasive biopsies. Several studies reported that immuno-PET with ^68^Ga, ^64^Cu or ^89^Zr could allow non-invasive assessment of lesions that are likely to respond to therapy [62,63,64,65]. In the ZEPHIR study, pre-treatment PET using ^89^Zr-labelled trastuzumab was explored in HER2-positive metastatic breast cancer patients scheduled for treatment with trastuzumab emtansine (T-DM1) [66]. PET using [^18^F]-fluoro deoxy glucose (FDG-PET) was performed at baseline and before the second cycle of T-DM1. Immuno-PET with anti-HER2 was negative in 29% of the studied population. Positive predictive value and negative predictive value were respectively of 72% and of 88% for immuno-PET and of 96% and of 83% for FDG-PET. Predictive values of 100% were obtained by combining immune-PET and FDG-PET, and patients with a time-to-treatment failure of 2.8 months from those with a time-to-treatment failure of 15 months were discriminated. This innovative work and results support the benefit of combining imaging methods to assess target expression with evaluation of drug impact on the target. To our knowledge, this is the first time such a combined approach has been undertaken and it clearly demonstrates its accuracy in predicting whether adequate tumor targeting is followed by sufficient efficacy and cytotoxicity.

The programmed cell death protein 1 (PD1)/programmed death-ligand 1 (PD-L1) axis is an important immune checkpoint for T cell activation. PD-L1 overexpression is associated with a poorer prognosis in a variety of cancers, especially in breast, gastric, renal cell, ovarian, non-small lung, hematologic cancers and melanoma [67,68,69,70,71,72,73]. Patients with PD-L1 overexpression typically have a stronger response to anti-PD-L1 therapy, such that determining PD-L1 expression allows identifying patients who will respond to anti-PD-L1 therapy. Additionally, monitoring changes in PD-L1 expression could provide information considering treatment efficacy or potential toxicity. The PD-L1 expression is usually evaluated using immunohistochemistry (IHC) on archive tissue samples, and does not consider that PD-L1 expression may undergo changes due to alterations in tumor microenvironment or previous treatment. Some preclinical studies showed the potential interest of noninvasive SPECT or PET imaging of tumor PD-L1 expression using radiolabelled anti-PD-L1 antibodies [74,75,76]; yet, to our knowledge and according to Clinicaltrials.gov, only one clinical study is under recruitment. The latter aims to evaluate the potential interest of an anti-PDL1 antibody called MPDL3280A labelled with ^89^Zr for non-invasive imaging and quantification of PD-L1 distribution in patients with locally advanced or metastatic non-small cell lung cancer, bladder cancer or triple-negative breast cancer.

Immuno-PET could provide information about tumor targeting, pharmacokinetics and accumulation in critical normal organs for RIT planning. By translating tumor-to-background ratios into potential absorbed radiation doses, this approach allows for improved optimal dosing. Impact of preloading with unlabelled antibody could also be assessed [77,78]. Rizvi et al. [79] conducted a prospective clinical study to evaluate the biodistribution and radiation dosimetry of ^90^Y-ibritumomab tiuxetan (Zevalin^®^) using ^89^Zr-ibritumomab tiuxetan. Patients with relapsed B-cell NHL underwent PET scans after injection of ^89^Zr-ibritumomab tiuxetan and again 2 weeks later after coinjection of ^90^Y-ibritumomab tiuxetan. Biodistribution of ^89^Zr-ibritumomab tiuxetan was not influenced by simultaneous treatment with ^90^Y-ibritumomab tiuxetan. High correlation was observed between predicted pre-therapy and absorbed therapy organ doses as based on ^89^Zr-ibritumomab tiuxetan images. These results are similar to previous data presented by Perk et al. [80], and confirm the potential value of pre-therapy ^89^Zr-immuno-PET to enable individualized treatment by optimizing RIT dose schedules for patients.

## 5. Conclusions

Today, the field of PET molecular imaging is rapidly progressing toward clinical use, providing repeatable non-invasive whole-body biomarkers mapping. Furthermore, immuno-PET represents a promising tool for personalized medicine in the context of multimodality treatment strategies. Consistent preclinical and clinical studies have been performed showing the safety, the improved image quality, as well as the potential for proper estimation of the antigenic expression level of immuno-PET. Moreover, it is attractive for studying the in vivo behavior of antibody-based therapies and for better understanding their therapy efficacy.
